# Supplementary material for: An ARMS-Multiplex PCR Targeting SARS-CoV-2 Omicron Sub-Variants
Source: Pathogens. 2023 Aug 6;12(8):1017. doi: 10.3390/pathogens12081017 (PMC10459702; doi:10.3390/pathogens12081017)
Supplement: Supplementary file 1 [file pathogens-12-01017-s001.zip › Supplementary Material Table S1.pdf]

**Characteristic SNPs within sub-variant group**	**SNP POSITION ACCORDING TO WUHAN SEQUENCE (NC_045512.2)**				
	**BA.1**	**BA.2**	**BA.3**	**BA.4**	**BA.5**
C -> T	-	-	832	-	-
A -> G	2832	-	-	-	-
G -> A	8393	-	-	-	-
C -> T	-	9866	-	-	-
C -> T	-	-	11235	-	-
A -> G	11537	-	-	-	-
T -> C	13195	-	-	-	-
C -> T	15240	-	-	-	-
T -> G	22195	-	-	-	-
T -> G	22197	-	-	-	-
A -> C	22198	-	-	-	-
C -> A	22202	-	-	-	-
T -> C	22204	-	-	-	-
T -> C	22672	-	-	-	-
G -> A	23048	-	-	-	-
C -> A	23202	-	-	-	-
C -> A	24130	-	-	-	-
C -> T	24503	-	-	-	-
G -> A	-	-	-	-	26529
G -> T	-	-	-	27788	-
C -> T	-	-	-	-	27889
C -> T	-	-	29311	-	-
